# Application of Deep Learning Techniques for Detection of Pneumothorax in Chest Radiographs

**DOI:** 10.3390/s23177369

**Published:** 2023-08-24

**Authors:** Lawrence Y. Deng, Xiang-Yann Lim, Tang-Yun Luo, Ming-Hsun Lee, Tzu-Ching Lin

**Affiliations:** 1 Department of Artificial Intelligence, Tamkang University, Tamsui, New Taipei City 251301, Taiwan; 114722@mail.tku.edu.tw; 2 Department of Computer Science and Information Engineering, Tamkang University, Tamsui, New Taipei City 251301, Taiwan; 811415016@o365.tku.edu.tw (X.-Y.L.); 805410056@gms.tku.edu.tw (T.-C.L.); 3 Office of Physical Education, Tamkang University, Tamsui, New Taipei City 251301, Taiwan; 159760@mail.tku.edu.tw; 4 Department of Radiology, Lotung Poh-Ai Hospital, Yilan 265501, Taiwan

**Keywords:** artificial intelligence, machine learning, X-ray, magnetic resonance imaging, Detectron2, lung diseases classification, image recognition

## Abstract

With the advent of Artificial Intelligence (AI) and even more so recently in the field of Machine Learning (ML), there has been rapid progress across the field. One of the prominent examples is image recognition in the medical category, such as X-ray imaging, Computed Tomography (CT), and Magnetic Resonance Imaging (MRI). It has the potential to alleviate a doctor’s heavy workload of sifting through large quantities of images. Due to the rising attention to lung-related diseases, such as pneumothorax and nodules, ML is being incorporated into the field in the hope of alleviating the already strained medical resources. In this study, we proposed a system that can detect pneumothorax diseases reliably. By comparing multiple models and hyperparameter configurations, we recommend a model for hospitals, as its focus on minimizing false positives aligns with the precision required by medical professionals. Through our cooperation with Poh-Ai Hospital, we acquired a total of over 8000 X-ray images, with more than 1000 of them from pneumothorax patients. We hope that by integrating AI systems into the automated process of scanning chest X-ray images with various diseases, more resources will be available in the already strained medical systems. Our proposed system showed that the best model that is used for transfer learning from our dataset performed with an AP of 51.57 and an AP75 of 61.40, with accuracy at 93.89%, a false positive of 1.12%, and a false negative of 4.99%. Based on the feedback from practicing doctors, they are more wary of false positives. For their use case, we recommend another model due to the lower false positive rate and higher accuracy compared with other models, which in our test shows a rate of only 0.88% and 95.68%, demonstrating the feasibility of the research. This promising result showed that it could be utilized in other types of diseases and expand to more hospitals and medical organizations, potentially benefitting more people.

## 1. Introduction

Pneumothorax is a disease that can be caused by multiple different factors, which leads to a cavity between the lung and the chest wall, which is also known as a collapsed lung. It occurs when air escapes from the envelopment of the lung. The symptoms of pneumothorax are sudden chest pain, shortness of breath, and blue-colored lips (cyanosis), which can be caused by hypoxemia. Pneumothorax can also be caused by gun or stab wounds or some other chest traumas [1]. There are around 17 patients per 100 thousand people, and over half of the afflicted (11 out of 100,000) will be hospitalized [2]. Compared with other types of lung diseases, pneumothorax needs to be identified and given care more urgently [3]. Treatment can be done from multiple aspects, from the severity of the disease. Spontaneous pneumothoraxes are more often than not regarded as harmless if the size is smaller than 50% of the hemithorax, the patient does not experience breathlessness, and there is no underlying lung disease [4]. Regarding the disease’s spontaneous nature, it sometimes is able to automatically resolve by itself. A larger area with breathlessness symptoms shown can be treated with aspiration [1]. Aspiration decreases the size of the cavity in most of the patients treated (>50%), reducing hospital admissions without increasing the risk of complications [5]. In other cases, it can also be treated with a chest tube treated with anesthesia [5]. Due to the circumstances mentioned above, although the difficulty of detecting pneumothorax by using AI is considered more difficult compared with other types of diseases, we decided to focus on it first in our research, heeding the advice of a professional doctor. Other types of diseases will be tried at a later time. Convolutional Neural Network (CNN) has already achieved remarkable results in CT imaging segmentation and proved to be even more successful in traditional detection in recent years [6,7]. In 2017, Meta Research (then Facebook AI Research, FAIR initiative) introduced software that implemented various object detection and segmentation algorithms, including Faster R-CNN, RetinaNet, and Mask R-CNN. Various research groups are currently utilizing this software for their research in medical imaging.

In the present stage, we proposed a deep learning model that identifies pneumothorax and gives a clear label to the chest X-ray image annotated with confidence values that can be used by medical professionals to obtain a grip on the usability and rough area of the disease. Our dataset was built from chest X-ray images taken from a hospital’s database and manually labeled before being rechecked and verified again by doctors from the hospital.

Our main contributions to this research are as follows:

- Various pneumothorax models are also augmented with multiple techniques that are available for download [8].
- A comparison between different parameters of a model.
- A comparison of various implementations of models.
- Metrics (high accuracy, low false positives) that are applicable to the medical imaging sector.
- The potential for transfer learning to other diseases.

The rest of the paper is arranged as follows. Section 2 discusses previous research in Machine Learning and deep learning in X-ray image and MRI segmentation. Section 3 introduces the dataset used for training the model, the architecture, and the workflow. The result analysis of the proposed model is conferred in Section 4. In Section 5, we draw conclusions based on the finding of this research and discuss potential avenues for future research and work.

## 2. Related Works

We utilized a method frequently used in medical imaging called Mask R-CNN [9], as recommended by a professional doctor, and transferred training upon a modified ResNet [10] to train and build an image recognition system that can be used to identify different diseases by using X-ray images, which can help doctors on diagnosing pneumothorax and various other diseases. With the assistance of image recognition technology, we hope to minimize the time needed for general practices and, with that, decrease the maintenance cost of staff and increase the throughput of medical care attended. Moreover, this will help doctors to understand the root cause of diseases faster, more precisely, and correct snap decisions can be made, thus improving the overall medical quality. The research can be used as a basis for future research and development and to improve the model of image recognition in this field.

We hope to help doctors diagnose a patient faster and increase the detection rate of chest-related diseases. We also aim to assist doctors in making a more informed decision on diseases by decreasing the time doctors need to collect information. Our research is a project on building a lung disease X-ray imaging recognition system by utilizing AI. The system aims to offer benefits to the healthcare system overall because doctors can save time that also could be spent instead on tests, consultations, and treatments.

Of the vast landscape of research available to us, we can gain inspiration from a plethora of different methods and types of diseases being tried, and so we briefly review some of the common methods used in the below section.

Machine Learning was applied in many types of medical fields, such as pharmaceutical research. One such application was on the targeting of NLRP3 protein by Ishfaq and colleagues [11].

Another method is by using different approaches to the data inputs, such as by utilizing Ribonucleic acid (RNA) sequencing that generates RNA expression levels to send to a classifier algorithm [12], using segmented structures and extracting attributes from lung images as a basis of the classification step [13], or simply labeling multiple types of diseases as simply sick or healthy [14].

Chronic Obstructive Pulmonary Disease (COPD) will become the third leading cause of death worldwide by 2030 from the estimation of the World Health Organization; thus, Ramalho et al. [13] and A. Poreva et al. [14] are tackling these problems with different results, with various degrees of successes, with the paper from A. Poreva et al. even trying five different types of methods to determine which will be the better choice. They used 134 patients’ data for their research and obtained accuracy results ranging from the worst of 53% on logistic regression to the best of 88% on an SVM classifier, and Ramalho et al. [13] managed an accuracy of 79% and 85% on the two methods they have used. COPD patients sometimes also manifest a checkup if the patient occurred a breathlessness effect, as it might be a case of pneumothorax, too [5].

Another aspect of the use of ML in medical imaging detection is for brain tumors. According to the World Health Organization’s International Agency for Research for Cancer 2020 World Cancer Report, cancer of the brain and central nervous system was the 17th most common cancer type, with an estimated 297,000 new cases worldwide [15]. Often than not, the 5-year relative survivability of the infected is estimated at around 33% according to the SEER database [16]. Thus, there were various research groups targeting this aspect of ML.

K. Sharma et al. [17] used a method that converted MRI imagery to black and white before extracting features based on texture, while J. Amin et al. [18] used a combination of statistical and ML methods to achieve a high precision rate ranging from 88% to 97% on their proposed method. G. Hemanth et al., on the other hand, tuned the convolution mask to better retain the features of the images and reached a 91% accuracy [19]. Aamir et al. [20] increased the visual quality of MRI images by using a low-complexity algorithm before segmenting pneumothorax images.

The journey of our implementation started with the definitive paper of R-CNN [21]. R. Girshick et al. proposed a concept called Region Convolutional Neural Network (R-CNN), in which the region meant classify segments of an image instead of the whole image, and that yielded a more accurate picture of the local sector, which was advantageous in providing the local maxima in the accuracy of classification tasks. Their work at that time surpassed all other methods and everyone’s expectations and broke through the stalemate of detection in ML before, which also achieved results that were not only more accurate but also faster due to the locality nature.

Fast forwarding a year later, the same team provided the world with a follow-up over the previous R-CNN, calling it Fast R-CNN [22]. Fast R-CNN improved upon R-CNN by increasing the training speed over R-CNN by 9× and decreasing the training pipeline to single-stage. This allowed more features to be packed into the network and, in turn, also pushed Moore’s law by a few stages if no improvement had happened in the ML scene for the next few years, further improving the viability of ML.

Faster R-CNN [23] by S. Ren et al. improved upon Fast R-CNN by introducing a new network called Region Proposal Network (RPN), which shares its convolutional features with the detection network, resulting in cost-effective region proposals. RPN is a fully convolutional network that simultaneously predicts object bounds and objectness scores at each position. By combining the detection from Fast R-CNN and RPN, a Faster R-CNN further improved the accuracy of the network.

The main network we used in this research is based on a framework called Mask R-CNN [9] developed by He et al., and the aforementioned Faster R-CNN is authored partially by He, too. Mask R-CNN proposed a model that can classify objects and segment those said detected objects that are called the Region of Interest (RoI) at the same time on top of the improvement on RPN. The biggest impact it had on our research is that by reducing the classification volume, the computational difficulty is drastically decreased, thus lowering the bar for entering image classification and image segmentation.

Pneumothorax can also be caused by gun or stab wounds or some other chest traumas [1]. There are around 17 patients per 100 thousand people, and over half of the afflicted (11 out of 100,000) will be hospitalized [2]. Compared with other types of lung diseases, pneumothorax needs to be identified and given care more urgently [3].

## 3. Methodology

**Figure 1:**
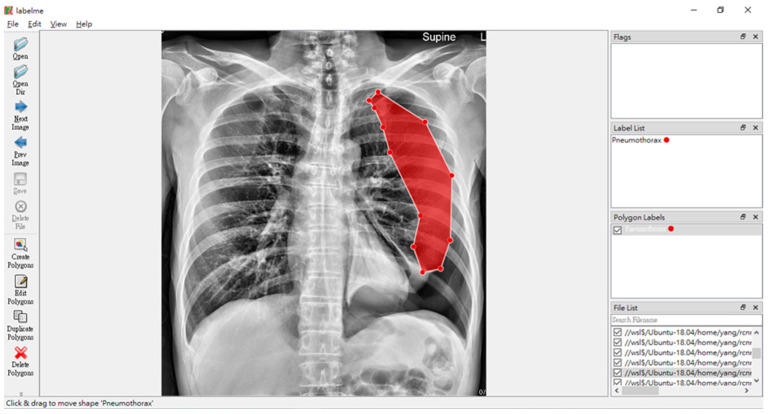
LabelMe in action on annotating pneumothorax, with the red shadow marking the ground truth of the affected area of the pneumothorax disease.

This research project aims to build a usable X-ray images lung disease detection system. Our dataset consists of over 8000 X-ray images, with more than 1000 of them from pneumothorax patients. In this project, we utilized a software called LabelMe [24], as shown in Figure 1. LabelMe annotation project is a standard software that is used to annotate interest segments from a given image and to classify those said segmented images.

**Figure 2:**
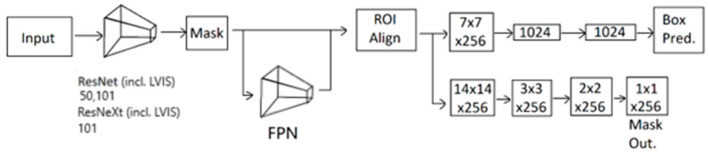
Process of the transfer learning of X-ray image detection system.

After annotation, the images were rechecked by the hospital’s doctors, and passing images made their way into the dataset, while rejected images were handled on a case-by-case basis with the doctors. Then, we resized the images. It was set to a maximum size of 800 on either side, whichever was the longest. After that, we chose model candidates for transfer learning. We used Detectron2 [25] in our research to detect pneumothorax because it is open source and quite useful in our endeavor. The main model choices were separated into two categories, which are ResNet [10] and ResNeXt [26]. These are the choices available on Detectron2. For ResNet, we used and compared model baselines that used Feature Pyramid Network (FPN) [27] because their COCO [28] instance segmentation baseline contained the best mask AP results; thus, R50-FPN and R101-FPN were chosen for the comparison. For ResNeXt, the only option was X101-FPN. Furthermore, we also compared the COCO dataset [28] implementation to the LVIS dataset [29] implementation for the respective ResNet50 (R50), ResNet101 (R101), and ResNeXt101 (X101) models. For both selections, they were first given to the model candidate to process to mask and later to FPN. Combining those, we will get various segments which are processed using Region of Interest Align (RoI Align). After the synchronization, this is separated into 2 networks, one for bounding box prediction and the other for mask segmentation for respective results. In this research, only the mask segmentation part will be relevant because the bounding box is not the final step and is not to our concern. The process of the transfer learning of the X-ray image detection system is as in Figure 2.

The training set consists of 784 images of pneumothorax instances. We ran the test with multiple different configurations, and for comparison, we laid out the results, too, in Table 1. These configurations are all from the R101-FPN baseline because X101 requires 192% of the time to run compared with R101, and R50 is more inaccurate compared with R101 and only saves 34% of the time [30]. We chose the 26-1501 config as the baseline due to the low false positive rate and accuracy. The selected config was as follows: 100,000 iterations, the learning rate was 0.001, the weight decay was 0.0001, and the batch size per image was 512. The decay steps, which were for each target iteration, and the learning rate would be decreased to 0.1× of the previous value, were 25 k, 40 k, 50 k, 60 k, 70 k, 80 k, 85 k, 90 k, and 95 k. This step was to slowly transition the descent to fine-tune the gradient.

When the best hyperparameter was contributions to this research are models. The candidates are as follows: ResNet50 (COCO), ResNet101 (COCO), ResNeXt101 (COCO), ResNet50 (LVIS), ResNet101 (LVIS), and ResNeXt101 (LVIS).

Our training with all the configurations was paired with various augmentations to the X-ray images to reduce the probability of overfitting during the session. The parameters of the augmentations were as follows:

- Rotation is set to be randomly shifted by 5 degrees.
- Brightness is set to be randomly tuned in a 20% range on the original luminosity of the image.
- Contrast is set to be randomly tuned in a 20% range of the original image.
- Saturation is set to be randomly tuned in a 20% range of the original image.

From observation, our test with four images per batch used around 10.2 GB of VRAM in the process. Without augmentations, five images per batch can be achieved for 10.8 GB. We used an RTX 3080 Ti, and 100 k iterations took 9 h and 30 min to complete.

## 4. Results

After training, the best results were dependent on the metrics. For false positives and accuracy, the best model is ResNeXt101 (COCO), while ResNeXt101 (LVIS) is the best for false negatives. ResNet101 (LVIS) performs best by a larger margin in both AP and AP75, with ResNet50 (LVIS) on AP50. The AP metric is from calculating the average between 10 precision-recall pairs from 50% to 95% with 5% increments in between. These are shown in Table 2.

**Figure 3:**
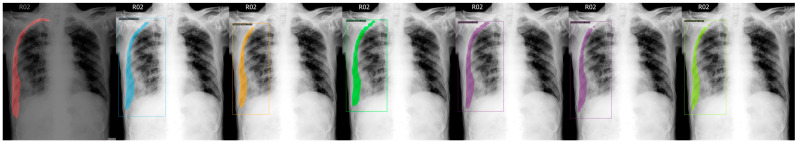
From left to right: Ground Truth, ResNet50 (COCO), ResNet101 (COCO), ResNeXt101 (COCO), ResNet50 (LVIS), ResNet101 (LVIS), and ResNeXt101 (LVIS). This patient had pneumothorax in their right chest.

**Figure 4:**
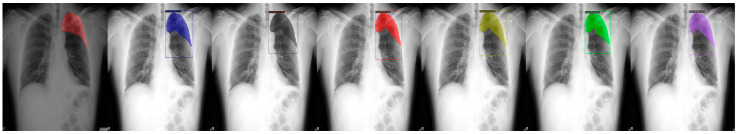
From left to right: Ground Truth, ResNet50 (COCO), ResNet101 (COCO), ResNeXt101 (COCO), ResNet50 (LVIS), ResNet101 (LVIS), and ResNeXt101 (LVIS). This patient had pneumothorax in their top-left chest.

**Figure 5:**
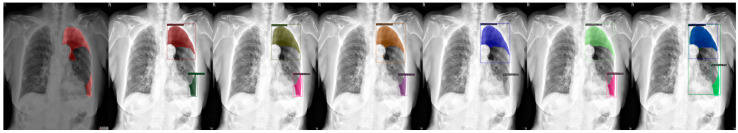
From left to right: Ground Truth, ResNet50 (COCO), ResNet101 (COCO), ResNeXt101 (COCO), ResNet50 (LVIS), ResNet101 (LVIS), and ResNeXt101 (LVIS). This patient had a large area of pneumothorax in their left chest.

**Figure 6:**
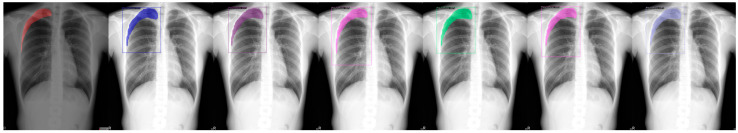
From left to right: Ground Truth, ResNet50 (COCO), ResNet101 (COCO), ResNeXt101 (COCO), ResNet50 (LVIS), ResNet101 (LVIS), and ResNeXt101 (LVIS). This patient had pneumothorax in their right chest.

**Figure 7:**
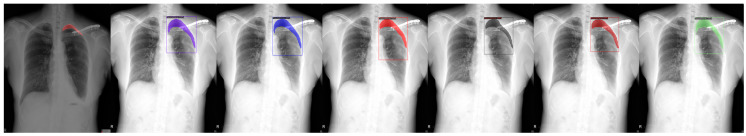
From left to right: Ground Truth, ResNet50 (COCO), ResNet101 (COCO), ResNeXt101 (COCO), ResNet50 (LVIS), ResNet101 (LVIS), and ResNeXt101 (LVIS). This patient had pneumothorax in their left chest while having a fixation due to a probable accident.

Below are the output results for the test images set, from Figure 3, Figure 4, Figure 5, Figure 6 and Figure 7, which are not in the training or validation set used for training the models.

We chose a few examples to demonstrate the capabilities of various models. These images consisted of X-ray images of pneumothorax patients with different areas of disease inflicted. The inclusion of multiple positions and areas in the figures lets us observe the possible outcomes and the differences between the models. In Figure 3 and Figure 6, the patients had pneumothorax on the right side of their chest, with the patient in Figure 6 only affecting the top part, while the patient in Figure 3 had pneumothorax on the outside wall of their right lung. In Figure 4, Figure 5 and Figure 7, the patients had pneumothorax in the left side of their chest, while the patients in Figure 4 and Figure 5 had the upper part of their left lung mostly collapsed, indicating a serious issue of pneumothorax disease. In Figure 5, there are cavity areas at the bottom part, too. In the same image, the ground truth included a portion protruding below the area of the top detection portion due to the late-stage pneumothorax with most of the upper part of the lung collapsing, and all models were unable to detect the cavity of the protruded part. In Figure 7, the patient is seen with a fixation, with the pneumothorax affecting the top left lung, albeit with a smaller area.

## 5. Discussion

In this paper, we used ML to detect pneumothorax with promising results. First, the collected images were preprocessed by resizing. Various hyperparameter configurations are tested for the best results. There were multiple options to go for, namely iterations, learning rate, decay steps, and weight decay. Iterations affect the final outcome, including false positives, false negatives, and overall accuracy. However, iterations that are too high will lead to overfitting. This can be partially mitigated by setting a stopgap measure after the accuracy has not been improving for a while. We recommend 100 k iterations for 1 k images with augmentations in consideration. The learning rate dictates the speed at which the learning occurs. Too small and the learning will take a long time, while too large will diverge the loss rate. We recommend a value of 0.001 for an adventurous exploration phase for the beginning and stepping down later to a fine-tuning phase. Decay steps decrease the learning rate by a factor of 10, slowly transitioning the model to fine-tuning the model itself. Our best results emerged from nine decay steps, occurring from 25 k to 95 k. Weight decay improved the overfitting issue aforementioned in the iterations part, but too high will lead to the learning rate being too slow or diverging outright. We recommend 0.0001 while increasing the decay steps count. After selecting the hyperparameter combination with the suitable results, which were dictated by the overall accuracy and AP with our choice of configurations, we used that config to train against different baseline models with the same X-ray images. After training, we picked our model following two criteria, AP (Average Precision) and false positives. AP was chosen due to it being the default metric recommended by the Detectron2 repository in their codebase. Secondly, the false positives metric was chosen based on the feedback from practicing doctors, who were more wary of false positives. Our proposed system showed that the best model after transfer learning from our study is ResNet101 (LVIS), with an AP of 51.57 and an AP75 of 61.40. The accuracy of the detection is 93.89%, with a false positive of 1.12% and a false negative of 4.99%. For the doctors’ use case, we recommend ResNeXt101 (COCO) due to the lower false positive rate and higher accuracy compared with other models, which in our test shows a rate of only 0.88% and 95.68%.

The research showed promising results, while more data will be needed for accurate comparison, which in the current state are more prone to overfitting the model and later to the possibility of utilization of the X-ray images on various different lung diseases, and eventually to a detection system to be used in the real world.

The current dataset consisted of data from our local hospital only, so we hoped to expand the dataset by cooperating with other hospitals from various areas around Taiwan. We are also discussing the possibility of including X-ray images from different machine models and manufacturers. Stronger machines are more cheaply available with each passing day. We can expand our resources for a faster training routine and a larger model to work with. With our experience gained in this research, we look forward to expanding the usage of this model type into other lung diseases or even other illnesses, providing a better future in the healthcare sector.

## Figures and Tables

**Table 1:** Various configurations and their respective results.

Configuration Candidate	Iterations	Learning Rate	Weight Decay	Decay Steps	False Positive (%)	False Negative (%)	Accuracy (%)	AP (%)	AP50 (%)	AP75 (%)
22-0710	37,500	0.1	0.005	1 k, 2 k, 5 k, 10 k, 15 k, 20 k, 30 k	3.16	9.41	87.43	**48.15**	**81.89**	46.17
23-1518	500,00	0.1	0.005	1 k, 2 k, 5 k, 10 k, 15 k, 20 k, 30 k	3.11	11.24	85.65	47.55	81.06	45.98
23-2251	50,000	0.1	0.005	500, 10 k, 20 k, 30 k, 35 k, 40 k, 45 k	2.97	11.68	85.35	43.25	78.90	43.05
24-1814	50,000	0.01	0.005	5 k, 10 k, 15 k, 20 k, 25 k, 30 k, 35 k, 40 k, 45 k	3.29	16.18	80.53	43.67	78.71	41.99
26-1501	100,000	0.001	0.0001	25 k, 40 k, 50 k, 60 k, 70 k, 80 k, 85 k, 90 k, 95 k	**1.09**	**3.68**	**95.23**	46.82	76.61	**50.46**

**Table 2:** Various models and their respective results.

Transfer Learning Model	False Positive (%)	False Negative (%)	Accuracy (%)	AP (%)	AP50 (%)	AP75 (%)
ResNet50 (COCO)	1.33	4.38	94.29	48.76	80.34	53.35
ResNet101 (COCO)	1.09	3.68	95.23	46.82	76.61	50.46
ResNeXt101 (COCO)	**0.88**	3.44	**95.68**	44.61	78.95	49.20
ResNet50 (LVIS)	1.47	5.91	92.62	49.26	**84.20**	53.45
ResNet101 (LVIS)	1.12	4.99	93.89	**51.57**	79.46	**61.40**
ResNeXt101 (LVIS)	1.05	**3.40**	95.55	49.50	82.20	50.79

## Data Availability

The model generated in this study is available for download at [https://github.com/xiangyann/PneumothoraxModel2023/releases/latest (accessed on 16 August 2023)].
